# Coming from behind to win - A Qualitative research about psychological conditions of adolescents who have undergone open-heart surgery for single ventricle between the ages 0-5

**DOI:** 10.1186/1749-8090-6-155

**Published:** 2011-11-23

**Authors:** Oguzhan Zahmacioglu, Cenk Eray Yildiz, Bulent Koca, Murat Ugurlucan, Selman Gokalp, Gurkan Cetin, Ayse Guler Eroglu, Funda Oztunc

**Affiliations:** 1 Department of Child and Adolescent Psychiatry, Yeditepe University Medical Faculty, Istanbul, Turkey; 2 Department of Cardiovascular Surgery, Istanbul University Institute of Cardiology, Istanbul, Turkey; 3 Department of Pediatric Cardiology, Istanbul University Cerrahpasa Medical Faculty, Istanbul, Turkey; 4 Cardiovascular Surgery Clinic, Duzce Ataturk State Hospital, Duzce, Turkey

**Keywords:** Congenital heart disease, Single ventricle, Fontan operation, Coping styles, Defense mechanisms; Experiences

## Abstract

Early recognition of congenital cardiac pathologies and their treatment by means of palliative or corrective surgery at birth or infancy has vital importance. Successful repair of congenital cardiac defects by surgical methods has gained importance especially during the last twenty years. As the scope of the surveillance increased so did the interest in the outcomes of these treatments when the patients had reached puberty and adulthood. The purpose of our research was to study the psychological framework of the adolescents who had experienced these surgeries by listening both the children and the parents talk about their feelings and experiences. Our data was accumulated through interviews with 17 adolescents and their families, using qualitative methods. The main theme at the end of the analysis was "to be strong and resistive". We reached the conclusion that this condition was not a pathological build up but an attitude of coping, as it did not cause loss of functionality. The defensive psychological mechanisms used by these adolescents consisted of repression, compensation and reaction formation. We believe that this information is important to understand the real meaning of the manners displayed when these adolescents and their families pursue their daily lives, communicate and make relationships with their environment and especially professionals in the health services.

## Introduction

Apprehensions of the parents when their children have health problems of vital importance have been widely witnessed. The natural unpredictability harbored by the possibility of losing a child is one of the most difficult trials encountered by the human mind. Some children and parents overcome this tribulation by the help of physicians, but some cannot. Early recognition of complex cardiac pathologies and their correction at birth or at infancy is therefore of vital importance. Life goes on for the lucky children who have undergone a successful surgery and the parents who have suffered profound anxiety throughout the entire process. On account of the developments in cardiac surgery, surveillance of the children with congenital cardiac defects has widened considerably enabling 85% of the cases to reach adolescence and adulthood [1]. Thus, the population of adolescents and young adults who have had open heart surgery at a very early age is increasing [2]. However, another process takes start in the lives of these individuals who have undergone surgery, such as periodic follow-ups and occasions of facing complications like arrhythmias, pulmonary hypertension, hematological problems and cardiac failure [3]. The questions are how good the quality of life will be for these patients, what the qualitative effects of difficult experiences in early life will be in young adulthood, and what the extent of the psychological effects of the illness are. There are studies on the lives of adolescents and young adults who were born with congenital heart disorders. Some are designed to assess academic performance by means of tests and questionnaires and some investigate quality of life, anxiety status, depression and conformity [4-7], and have been useful in revealing the psychological problems experienced. There are relatively much less data on those who have had successful corrective surgery at a very early age and are able to survive without suffering major health problems. Reports of qualitative research on such cases are very rare [8]. There are also studies on children and adolescents not just with cardiac disorders but other chronic diseases giving information on the relationships with family and friends, school life, and physical mobility [9]. Comparative studies including healthy controls have reported the relatively increased susceptibility of these patients to psychiatric disorders [10-12]. However, some studies have shown no psychological differences between these children and healthy controls [13]. We have aimed to broaden the limited data available on the subject of this study.

## Materials and methods

### Qualitative research

Sampling for qualitative research is an area of considerable confusion for researchers who are experienced in the quantitative models. One reason of that is the samples for qualitative research tend to be small. In practice, the number of required subjects usually becomes obvious as the study progresses, as new categories, themes or explanations stop emerging from the data (data saturation) [14]. A qualitative sample must be big enough to assure that the readers are likely to hear most or all of the perceptions that might be important. As in the case of our study, the day we observed that the patients kept on repeating some patterns in the interviews, no more new patterns have emerged, we stopped the data collection period.

Qualitative research consists of data collection through observation, interviews and study of documents and aims at presenting perceptions and events in their entirety and realistically in a natural context. The findings of qualitative research offer not a cross sectional but a multilayered data in a special construct taken under magnification, and therefore cannot be generalized. Hence, refined facts are reached in a narrow field of investigation [15]. Qualitative and quantitative researches are no longer regarded as alternatives but as supplementary approaches.

In this study the method of the grounded theory has been used. This way, disease experience of experimental subjects and the dynamic interrelationships of these experiences are revealed with the aim to have a unified concept [16]. In our study 17 participants between the ages of 12 and 20, who had experienced at least once an open heart surgery between the ages of 0 and 5 years, were included together with their families. Only the patients with single ventricle who have had a Fontan operation were included in the study ([17] patients, 9 male/8 female, mean age: 19 yrs 6 months). Patient demographics were presented on Table 1. All of these patients were operated on cardiopulmonary bypass. Their current saturations were between 85-90% and NYHA status were all Class 2. By the Fontan operation, systemic venous return is directed to the pulmonary system and it is provided to sustain systemic circulation with a single ventricle. Patients who undergo Fontan type repairs are considered separately from other patients with congenital cyanotic heart diseases because of the metabolic and hormonal factors and problems such as low saturation rate. The pre-Fontan procedures which are applied to the patients were: balloon atrial septostomy in 2 cases, Glenn shunt in 9 patients, modified Blalock-Taussig shunt in 5 patients, 1 case of pulmonary band (Table 1).

**Table 1:** Patients demographics

Patient No	Cardiac anomaly	1st operation	Age of 1st operation	2nd operation	Age of 2nd operation
**1**	TA+PS+HRV	Atrial septostomi	1st day	Classical Fontan	1-2 years

**2**	TA+PS+HRV	Glenn shunt	0-1 years	Classical Fontan	1-2 years

**3**	TA+PS+HRV	Glenn shunt	0-1 years	Classical Fontan	2-3 years

**4**	TA+PS+HRV	Glenn shunt	0-1 years	Intracardiac tunnel	2-3 years

**5**	TA+PS+HRV	Glenn shunt	2-3 years	Extracardiac tunnel	3-4 years

**6**	TA+PS+HRV	Glenn shunt	1-2 years	Extracardiac tunnel	3-4 years

**7**	TA+PS+HRV	Glenn shunt	0-1 years	Extracardiac tunnel	3-4 years

**8**	TA+PS+HRV	Atrial septostomi	1 day	Extracardiac tunnel	4-5 years

**9**	IVSPA+HRV	Glenn shunt	0-1 years	Classical Fontan	1-2 years

**10**	IVSPA+HRV	Blalock-Taussig shunt	4 months	Classical Fontan	1-2 years

**11**	IVSPA+HRV	Glenn shunt	0-1 years	Extracardiac tunnel	1-2 years

**12**	IVSPA+HRV	Blalock-Taussig shunt	1-2 years	Extracardiac tunnel	2-3 years

**13**	DILV	Pulmoner band	1 month	Intracardiac tunnel	2-3 years

**14**	DILV	Glenn shunt	1-2 years	Intracardiac tunnel	2-3 years

**15**	DILV	Blalock-Taussig shunt	2-3 years	Intracardiac tunnel	3-4 years

**16**	DORV+PS+HLV	Blalock-Taussig shunt	1-2 years	Intracardiac tunnel	1-2 years

**17**	PA+VSD+HRV	Blalock-Taussig shunt	1-2 years	Intracardiac tunnel	2-3 years

**DILV: **Double-inlet left ventricle, **IVSPA: **Pulmonary atresia with intact ventricular septum, **DORV: **Double-outlet right ventricle, **HLV: **Hypoplastic left ventricle, **HRV: **Hypoplastic right ventricle, **PA: **Pulmonary atresia, **PS: **Pulmonary stenosis, **TA: **Tricuspid atresia, **VSD: **Ventricular septal defect.

These young people were selected with the principle of "result oriented sampling" [17], such that the age range and a linguistic ability with the sociocultural background to communicate with a semi-structured interview were the characteristics seeked. The participants were selected from the patients visiting the clinic during a period of one month for routine clinical follow-ups. Those fitting the criteria of the research were made familiar with the details of the program and were referred to the researchers together with their families. They were informed that there would be voice recording and asked to sign a consent form. The research program was approved by the institutional ethics committee (Decree no. D-004, Date: 11-10-2009).

Subsequently, the participants were met on different dates for the purposes of in-depth interviews conducted in peaceful surrounds of a special room at the clinics. To eliminate thematic and stylistic differences, all interviews were conducted by the same interviewer who had previously attended a comprehensive training program on qualitative interview techniques receiving a certificate as a qualified interviewer. Each interview lasted 50 minutes with the patients and 30 minutes with their families. In order to protect the identities of the participants pseudonyms were used. The interviews were tape-recorded.

### In-depth interviewing

This is a semi-structured technique that entails no previously prepared questions but only themes, which the participants are made familiar with when invited to discuss the aims of the research. The interviewer is in a state of "active passivity". The interview is not interrupted unless very necessary. If the participant deviates significantly from the aim of the interview, open ended questions relevant to the previously determined themes may be asked without interrupting the flow of the interview [18].

### Data analysis

After the interviews, the voice recordings were transcribed word by word to papers. To begin with, expressions repeated or resembling one another were identified and coded, and subsequently these coded parts were placed in groups for analysis.

Analytical categories were defined according to the previously determined themes of the interviews. When the transcript material was assigned to the defined categories, 6 different writers read the context in order to reach a consensus. The themes repeated by different participants were also specially evaluated for the potential to be generalized.

## Results

In our study the main theme of the psychological formation of adolescents, who had experienced surgical repair of congenital heart defects between the ages of 0 and 5 years, was "feeling resistive and strong". During the interviews, irrespective of the subject talked about, they kept emphasizing how "strong and resistant" they had been. A number of secondary factors, the most striking ones being relationships with family and close circles, personal temperament and medical history, determined the depth and the limits of the influence of this theme in their daily lives. Here, we have attempted to demonstrate this theme within the statements of the patients quoted from the transcripts.

### Being strong and resistive

This theme that gained prominence in our study was independent of the participants' age and gender. It could be said that the patients put forward loudly the strength of their individuality through the memories narrated, examples given and even the body language during the conversations. We frequently met repetitions of expressions like "naturally I have done it", "of course I have, shouldn't I have done it?", "I did it first", "they ask me to do it", and others similar to these. These challenging verbal expressions appeared in their daily lives and activities in very different ways. Some led lives that did not reflect the austerity of the language they used. This group consisted of the individuals who did not take risks, avoided physical exertion, controlled their impulses well and observed the regularity of their medical controls. The defensive manner that pervaded their language had not upset their functionality. One participant representing this group was a 16-year-old male who was successful in high school and sustaining good relationships with friends. He said: "*...When something needs to be done (around the house), they make warnings like 'don't touch, don't lift', which upset me though I know that they do this for my own good. Because, if I do something I never do less than what is necessary, in fact I do the extras! On the other hand, it is I who didn't want to join the physical education class. I think it is unnecessary. Instead, I sit and solve test questions which is much better"*.

An eighteen-year old young girl, working as a cashier in a supermarket, who knew she was unwell but had made peace with this situation, has remarked: *"I am satisfied with my work. It's a little tedious, that's all. Sometimes I help my friends as well. There are those who know that I am ill, but they swear that I am much healthier than they are, because I don't get tired easily... But I don't get into excesses; I don't lift heavy items... I don't idle at home either. I cook, do the dusting... I don't enjoy sitting about like that..."*

Another group, although employing a similar tenor of language, consisted of patients with very pronounced denial of illness that were found to be more prone to behavioral nonconformity. They were pretending to ignore the illness continuing to make its presence felt through difficult follow-ups. It was evident that talking about their illness or their feelings was difficult for them. A 19-year old young man attending a night school, and being regularly followed up after two operations in his childhood said: "*I remember my illness only once every six months, at the control times... (Smiling) And my mother reminds me of that; otherwise I'll forget all about it... I don't see any problems in me... Anyhow, I don't have any difficulties... I cannot even recall having cried once! But, if those around me or people I love get into trouble, it tends to get fixed strangely in my mind. I love people very much.... It hurts very sharply when I see the needy and the helplessness... I would help all of them if I could"*.

### Secondary factors

#### Family/close circle

The attitudes of family and the close circle of friends influenced positively and/or negatively the life of the patients. A congenital disease of vital significance that has presented early in the life of the offspring causes, next to profound worry about the outcomes, a feeling of guilt in the parents which is reflected in the exaggeration of an already inordinate protectiveness towards the sick child. One of the mothers who, despite the explanations given by the physicians, could not avoid thinking in this manner has said: *"At the beginning I cried for days for this befalling on us; worrying if I had done something wrong during my pregnancy, but couldn't reach at anything... There are cardiac patients in the family, and some did have cardiac failure, but I was told it had nothing to do with these... My husband was angry with me for not having been careful about my nutrition during my pregnancy... Those in my own family blamed me for having done too much house work and making myself very tired... I kept suspecting the use of antibiotics for 3 consecutive days... Everyone around took a blaming attitude towards me at the time.''*

This mother's 15-year old daughter, fed up by the protectiveness of her family, has remarked: *"Even rope jumping was forbidden. Sometimes we went behind the house with friends so that my mother wouldn't see me, and I got mad when they shouted at me from the balcony... I believe now that they were more restrictive than the physicians. What the doctors didn't but my parents did."*

Some families had changed their lifestyle permanently by moving from their hometowns to receive better health service. For example: *"When she was a baby, we shut shop and home and came to Istanbul. Everything was left behind and we had no money... We had to stay in a hospital... Her mother was very distressed that she might die any day... Then she had an operation and got better, but we settled here for her follow-ups. It had been more than 10 years, we miss our hometown but this is more important for us."*

This father of the 15-year-old kid, a successful high school student, who had grown up recognizing the sacrifices made for her, and had developed a perfectionist attitude feeling the need to return these favors. She said, *"I'm happy that my school performance is good, but could have been better. I cannot sleep at night feeling miserable if I got 4 instead of 5 (the top mark)... My parents never put pressure on me... They wouldn't say anything even if I got bad marks; they often advise me not to make myself very tired with overwork, but I do not feel satisfied... They have confidence in me and I do not want to disappoint them."*

What the father of a 14-year old girl, who was in normal development through follow-ups after an early surgery, said during the interview reveals the experiences of parents before surgery and through the dramatic turn of events afterwards. "*Those days, when she was not yet operated, she would go purple (hypoxic) all of a sudden... I wasn't saying anything to her mother but kept thinking that this child would not survive much longer...During the surgery we nearly died with worry, and cannot describe you our pain... And now you see the result..."*

The families of the group of adolescents who had been successfully operated and were continuing "normally" through the follow-ups had developed strong instincts to protect the ongoing normality. This protective and interfering attitude further aggravated the already present periodic frictions between the adolescents and the parents. The statements of the mother of a 17-year-old indicated how such attitudes or protective parenthood remain unaltered despite the passage of years. *"...Strong headed is our boy... drinks cold water when hot with sweat, never pays attention to what he eats, indulges in unhealthy things like hamburgers... One becomes the bad person for having warned him... He shouts at me... I don't have to warn my other son who takes better care of himself. It is A. who has to be careful on account of his sensitive constitution... May be he would hit you if *you *say something to him."*

### Personality/temperament and sociocultural factors

The defense mechanisms used and the mode of handling this chronic process of medical follow ups by the patients and the families were directly correlated with their personalities and temperaments. What was said about illness and the acceptance of becoming ill in their own cultural surrounds were also effective on these modes of behavior. This can be seen in what the father of a boy, who had reached healthy adolescence after 2 operations, narrated. He said *"We took it as God's decision. Nevertheless we were wretched when the doctors made the diagnosis. Yet we didn't question His wisdom, we didn't rebel. The very same God has also granted the cure, because the doctors were able to make my child good."*

A cheerful-faced and energetic young girl, who had been operated, commented as *"In fact I regarded myself not as unlucky but as someone special... If I have been able to stand up despite all that happened, then I must be special... I was seen as a sick person, and, yes, I am still being seen as one... But this has made me different. For example, in primary school my teacher used to watch over me, and I had friends who were envious of that."*

There have also been patients who, on grounds of their personality, reacted negatively to the cautious approach of the close circle of friends to their illness. One claimed as *"When I was little and about to do something, I got very irritated when I heard from those around the warning ''but you are ill'. I didn't give indications, though... They weren't meaning badly after all. But, I cannot remember now how many times I must have hit with my fists the wardrobe in my room when I got back home."*

### Medical history

As all the participants had been operated between the ages of 0 and 5 years, they had virtually no memory of the events before the surgery. They belonged to a lucky group who had been able to survive with the help of successful treatment by the healthcare professionals. Mostly what consciously recalled were scenes resembling the ongoing medical controls. They were regularly admitted to the clinics and those working there were naturally the most important objects in their histories. Feelings like affection, exasperation, respect, fear, familiarity, boredom which echoed in their conversations were unavoidable acquisitions of close and long term relationships.

The expressions about being appreciated, being favored and popularity used by a 19-year-old shop salesman, who had been operated at the age of 2, were striking: "*Doctors get surprised every time I go for control. They see me in perfect condition. Apparently I have been their best patient."*

On the other hand, the slightly angry expressions of the 17-year-old high school girl, who might have to be operated another time, reflected her anxiety. *"Thanks to the doctors for having tried very hard, but I also have heard that mistakes were made in the past... One naturally cannot help protesting at times... What upsets me most is the uncertainty of when it will all end."*

## Discussion

### "Being strong and resistive" - psychological defense mechanisms

We believe that the theme of "being strong and resistive" that emerged from our data could best be explained by some psychological defense mechanisms. We came to the conclusion that three psychological mechanisms, namely repression, compensation and reaction formation, were being used. According to the Freudian psychoanalytical theory, defensive mechanisms are subconscious processes restored in order to sustain one's self image and to cope with the disturbing realities of the external world [19]. The main objective is to avoid anxiety. Defensive mechanisms do not always indicate the presence of a psychopathological problem. Healthy people also form different defensive mechanisms; and, according to Anna Freud every individual has a repertory specific to his/her own self. Only if these are continually disruptive to psychological functionality, can they be identified as being pathological. According to Vaillant, defense mechanisms are divided into 4 main groups: pathological, immature, neurotic and mature [20]. We can look closely at the three mechanisms we have observed in our study from this point of view.

### Repression

This is the exclusion of memories and experiences from the conscious, and their maintenance there. It constitutes the basis of all defense mechanisms. The ego generally opposes the reappearance in the conscious of those memories, desires, motives and feelings pushed out of it. These have been banned by the superego after being judged as items giving pain and anxiety to the ego. They are of a neurotic nature and therefore repressed.

In the majority of the adolescents participating in our study, currently experienced health problems that had started early in life have been of traumatic and painful nature to be kept outside the conscious by the ego. In their words and body language there were little indication of the difficulties they had experienced. Probably, having been operated very early in life, there weren't definitive 'bad memories' and feelings in the conscious to mention. Also, they didn't seem to have been keen to hear their personal and medical history from the family circles, because there wasn't anybody relating what was heard from others about his/her illness during the course of our interviews. Furthermore, there had not been any references to the future or their expectations from life. In accordance with McMurray et al. the unknown and the uncertainty of their illness might have prevented the process of taking initiatives and making decisions [21]. The use of the repression mechanism by children and adolescents with health problems has only recently begun to be discussed in the literature [22]. Weinberger, in his article on the subject of repression, takes a different angle from that of the Freudian theory, by arguing that repression is used by children and adolescents especially against the risk of social rejection, and that it might become part of the personality while improving social conformity [23]. Where as some authors have recognized the repression mechanism as a subconscious process [24], others have seen it as a conscious and purposeful style of coping with internal (e.g., illness) and external stimuli [25].

### Compensation

Compensatory reactions are developed against feelings of inadequacy rooted in real or imaginary lacks of the individual, who tries to cover what seem failures or incompetence with what has been successful and has received recognition ([18,19]). For example, an individual with a disability can compensate the negative effects of this condition after continuous struggle. People without sight have been known to receive awards for painting. The efforts in the school or work place to gain prominence, the successes and the appreciation gained indicate that compensation mechanism is frequently used in the lives of the adolescents we have interviewed and serve a useful purpose. Some work in jobs or do sports that strain their physical limits as if to prove something, while others overwork for school and examination success. These observations are completely in accordance with those in other reports [26].

### Reaction formation

Here the drives and feelings that cause anxiety are turned in the opposite direction and get accepted by the ego. Thus, an individual who cannot cope with feelings of incompetence can be swung to feelings of extreme competence and display this in his attitudes. In our interviews, born with heart defects that necessitated surgery, we encountered extreme self-confidence and optimism instead of brittleness, anxiety, insecurity and pessimism, which are expected to be witnessed at first sight. To have their active illness terminated by surgery was naturally important, since apart from a few, they were not still experiencing cardiac concerns. But despite this reality, it was possible to observe in their tone of voice, their eyes and the words they selected the sharpness and the challenging manner natural to adolescence.

### Family and the close social circle

The helplessness and hopelessness experienced by the families of the participating patients in this study before their children underwent corrective surgery was still expressed during the interviews. These observations accorded with the findings of Lawoko and Soares [27] in the comparative studies with the parents of children suffering from other types of chronic diseases. Next to this, there were also parallelisms between our findings and those in other reports on the extreme protectiveness of the parents ([28,29]).

To be protected, watched over and treated preferentially by their families had become a routine for our interviewees. They did not much complain about this protective attitude, and even saw it as normal. But, as explained by Gersony *et al*. [30], to be prevented by the family from making efforts and to be shielded on grounds of being ''sick'' consolidated the feelings of inadequacy. Our findings have supported these conclusions.

### Medical history

The perception as father and mother figures of the doctors who had operated on them by the patients was one of the most significant observations in this study. The anger and protestations of the patients whose follow-ups progressed with more difficulty, referrals to the praising received by those who attended the follow-ups regularly, the desire to be the favorite are feelings compatible with those established by children with their parents.

In this study we have witnessed that, although operated at a very early age, the interviewed adolescents carried the traces of the past and ongoing medical processes in different layers of the conscious. The defense mechanisms used and the styles of coping generally did not involve any psychopathology, but, on the contrary, they served as a life buoy that enabled the continuation of their lives. As these attitudes that have tended to become part of their personality pattern is recognized, the physicians who take care of them can establish a closer and more straightforward relationship with this group of patients. This signifies for the doctor patient cooperation to ensure the correct progress of those illnesses requiring long-term follow-ups.

Apart from the clinical findings, we also think that next to standard measures and questionnaires of practical use, the method of gathering raw data through direct interviews with patients without any predictions should also be recognized in the health service. The most important advantage of this study has been the allocation of a 60-minute interview to each patient and a 30 minutes interview to each family.

### Limitations

The major limitation of the study is that the findings cannot be generalized given the nature of the research method selected. But we believe that these findings nevertheless would support further research of wider scope and deeper reach.

Another limitation regards to the small number of the patients. However, the research includes a specific patient population with congenital cardiac disease; i.e. the patients with single ventricle whom have undergone congenital heart surgery for the particular defect.

## Conclusion

Early recognition of congenital cardiac pathologies and their treatment by means of palliative or corrective surgery at birth or infancy has vital importance. Successful repair of congenital cardiac defects by surgical methods has gained importance especially during the last twenty years. As the scope of the surveillance increased so did the interest in the outcomes of these treatments when the patients had reached puberty and adulthood. We aimed to research was to the psychological framework of the adolescents who had experienced surgeries for single ventricle heart physiology. We listened both the children and the parents about their feelings and experiences. The main theme at the end of the analysis was "to be strong and resistive". We reached the conclusion that this condition was not a pathological build up but an attitude of coping, as it did not cause loss of functionality. The defensive psychological mechanisms used by these adolescents consisted of repression, compensation and reaction formation. We believe this information is important to understand the real meaning of the manners displayed when these adolescents and their families pursue their daily lives, communicate and keep relations with their environment and especially professionals in the health services.

## Competing interests

The authors declare that they have no competing interests.

## Authors' contributions

OZ, CEY, BK, SG, GC, AGE, and FO acted in conception and design. OZ, CEY, MU, and GC acted in data analysis and interpretation. OZ, CEY, and MU acted in manuscript writing. OZ, CEY, MU, and GC acted in revision of the article. All Authors read and approved the final Manuscript.
